# Delivery mode and altered infant growth at 1 year of life in India

**DOI:** 10.1038/s41390-021-01417-6

**Published:** 2021-03-02

**Authors:** Giridhara R. Babu, Noel T. Mueller, Melissa Glenda Lewis, Anjaly Krishnan, Eunice Lobo, R. Deepa, Sonalini Khetrapal, Sara E. Benjamin-Neelon

**Affiliations:** 1 Indian Institute of Public Health-Bengaluru, Public Health Foundation of India (PHFI), Bengaluru, India; 2 Wellcome Trust-DBT India Alliance Intermediate Research Fellow in Public Health, Hyderabad, India; 3 Department of Epidemiology, Johns Hopkins Bloomberg School of Public Health, Baltimore, MD USA; 4 Indian Institute of Public Health-Hyderabad, Public Health Foundation of India (PHFI), Hyderabad, India; 5 Asian Development Bank, Manila, Philippines; 6 Department of Health, Behavior and Society, Johns Hopkins Bloomberg School of Public Health, Baltimore, MD USA

## Abstract

**Background:**

Cesarean section (C-section) delivered infants are more likely to be colonized by opportunistic pathogens, resulting in altered growth. We examined whether C-section (elective/emergency) vs vaginal delivery was associated with altered weight and linear growth at 1 year of life.

**Methods:**

A total of 638 mother–infant pairs were included from MAASTHI cohort 2016–2019. Information on delivery mode was obtained from medical records. Based on WHO child growth standards, body mass index-forage *z*-score (BMI *z*) and length-for-age *z*-score (length *z*) were derived. We ran multivariable linear and Poisson regression models before and after multiple imputation.

**Results:**

The rate of C-section was 43.4% (26.5%: emergency, 16.9%: elective). Percentage of infant overweight was 14.9%. Compared to vaginal delivery, elective C-section was associated with *β* = 0.57 (95% CI 0.20, 0.95) higher BMI *z*. Also infants born by elective C-section had RR = 2.44 (95% CI 1.35, 4.41) higher risk of being overweight; no such association was found for emergency C-section. Also, elective C-section delivery was associated with reduced linear growth at 1 year after multiple imputation (*β* = −0.38, 95% CI −0.76, −0.01).

**Conclusions:**

Elective C-section delivery might contribute to excess weight and also possibly reduced linear growth at 1 year of age in children from low- and middle-income countries.

**Impact:**

- Our study, in a low-income setting, suggests that elective, but not emergency, C-section is associated with excess infant BMI *z* at 1 year of age and elective C (C-section) was also associated with altered linear growth but only in multiple imputation analyses.
- Elective C-section was associated with a higher risk of being overweight at 1 year of age.
- Our results indicate that decreasing medically unnecessary elective C-section deliveries may help limit excess weight gain and stunted linear growth among infants.

## Introduction

The double burden of overweight and obesity and undernutrition (e.g., stunting) continues to rise globally, affecting one-third of low and middle-income countries (LMICs).^1^ An estimated ≥2.28 billion overweight children and >150 million stunted children are in LMICs.^1,2^ Altered early growth may portend future risk of obesity,^3^ and as such identifying modifiable exposures during the first 1000 days—from conception to 2 years of age^4^—may help to inform the policies and interventions that are needed to address this global health challenge.

One exposure in the first 1000 days that has been associated with excess infant weight gain and childhood overweight and obesity is Cesarean section (C-section) delivery.^4^ While several mechanisms may explain this association, including differential stress response to labor^5^ and delivery-mode-induced epigenetic programming,^6^ the leading hypothesis is that interruption of mother-to-newborn microbiota transfer drives the association of C-section with overweight and obesity. Consistent with this hypothesis, studies have found that gut microbiota may play an etiologic role in the development of overweight, as well as undernutrition,^7^ and C-section is highly deterministic of the infant gut microbiota development.^8^ Moreover, the prevalence of C-section delivery is on the rise globally,^9^ including in countries like India^10,11^ that have a high double burden of malnutrition. Yet, while C-section has been associated with excess weight in US infants,^12^ no studies to our knowledge have examined whether C-section is associated with both infant overweight and stunted linear growth in a developing country.

Given these gaps in the literature, we examined the association of delivery mode (C-section vs vaginal) with BMI *z*-score and length-for-age *z*-score at 1 year in a longitudinal Indian birth cohort.^13^ We further assessed whether associations differed by C-section type (emergency vs elective), which has been shown to modify mother-to-newborn microbial transmission and microbiota-associated outcomes.^14–16^ We hypothesized that C-section is associated with higher infant BMI *z*-score and lower length-for-age *z*-score at 1 year and that associations are stronger for elective C-sections because of reduced mother–newborn microbial transmission.

## Methods

### Participants

We analyzed data from the Maternal Antecedents of Adiposity and Studying the Transgenerational role of Hyperglycaemia and Insulin (MAASTHI) prospective birth cohort. We previously published a detailed cohort protocol that included information on the study design and methodology.^13^ In brief, the MAASTHI cohort was designed to study maternal risk factors associated with childhood overweight and obesity. The cohort was initiated in April 2016. For recruitment, we approached pregnant women, between 14 and 36 weeks of gestation, who were receiving care from public health prenatal clinics in Bengaluru, Karnataka, South India. These public health clinics cater to women from lower and middle socioeconomic strata from the surrounding communities. We enrolled women who resided in the study area, had plans to deliver at one of the public health hospitals, and were having a singleton pregnancy without any congenital abnormalities. We excluded women with chronic conditions or illnesses (diabetes, Hepatitis B infection, HIV positivity) and women who were unable to undergo oral glucose tolerance tests (OGTTs) before 36 weeks of gestation. Informed consent was obtained from all participants, and the study was approved by the Institutional ethics committee (IEC) of the Indian Institute of Public Health-Bengaluru.

**Fig. 1:**
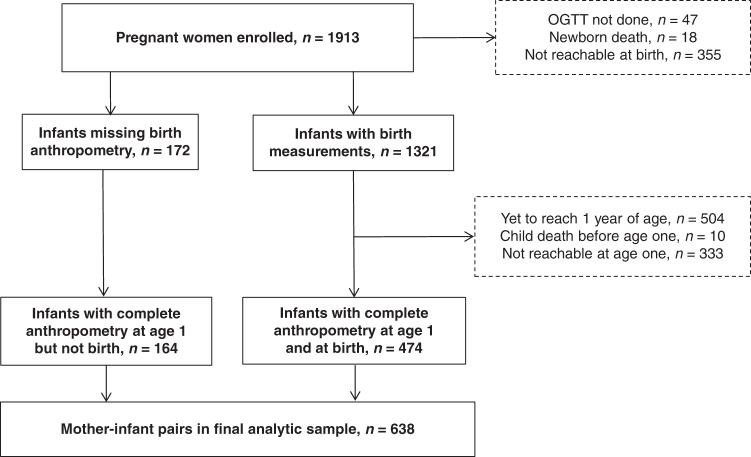
Recruitment and follow-up of study partcipants. Flow chart depicting the recruitment of study participants.

Data for the current analyses were collected from April 2016 to June 2019. A total of 1913 women delivered during this time. From these 1913 women, we excluded participants who did not deliver at the study hospital or were not available by phone (*n* = 355), who did not complete an OGTT in pregnancy (*n* = 47), and who had newborns who died during delivery (*n* = 18). Among the remaining 1493 mother–child dyad, 1321 had anthropometry recorded at birth, and another 164 had anthropometry recorded only at 1 year. Of the 1321 who had anthropometry recorded at birth, 504 infants had not completed 1 year of age and 474 were followed up and had anthropometry at 1 year. Among those who were lost to follow-up, 10 children had died, and 333 were not reachable by phone/physically. A total of 638 infants had completed anthropometry at 1 year, and they were included in the final analysis (Fig. 1).

### Exposure measurement

Our primary exposure of interest was the mode of delivery, which we documented using medical records. Vaginal delivery types included spontaneous vaginal delivery and operative vaginal delivery (via forceps). Due to the small number of operative vaginal deliveries, we combined operative (*n* = 5) and spontaneous vaginal delivery (*n* = 361) into a single category. C-section delivery types were emergency and elective. Emergency C-section was performed based on the medical condition of the mother, as decided by the consulting doctor. In contrast, elective C-section was when the pregnant woman chooses to deliver the baby through C-section delivery.

### Outcome measurement

We assessed infants’ anthropometry at two time points after delivery: within 7 days of birth and at approximately 1 year (300–500 days). Anthropometric assessments included measurement of weight (kg) and length (cm). For infant weight, we used a single reading to the nearest 0.5 g on a calibrated digital weighing scale (SECA 354) with minimal clothes on the infant. We calculated age- and sex-specific BMI *z*-scores (BMI *z*) and length-for-age *z*-scores (length *z*) using references charts from the WHO Child Growth Standards for infants aged between 0 and 60 months.^17^ This was done using the addWGSR() function of package “zscorer” in R (R Core Team, 2019).^18^ We considered infant BMI *z* at 1 year as a continuous measure, and we categorized infants with BMI *z* ≥85th percentile as being overweight.

### Other measures

During the baseline interview, women self-reported age, parity, religion, occupation, education, and household income. We categorized socioeconomic status using the Kuppuswamy scale of socioeconomic status, which takes into account education, occupation, and income.^19^ Based on Kuppuswamy scores, women were categorized as upper, upper-middle, lower-middle, upper-lower, and lower socioeconomic status. For analysis, we reclassified socioeconomic status into “lower” (consisting of upper lower and lower) and “upper” (upper, upper middle, and lower middle). We ascertained maternal height (cm) and maternal weight (kg) at the first prenatal visit. We measured maternal weight using a digital Omron HN-283 (Omron Healthcare Co., Ltd.) scale. Research staff placed the scale on level ground and asked women to remove their shoes and any heavy or outer layers of clothing. We documented two readings to the nearest 10 g. We used a stadiometer (SECA 213) to measure height to the nearest 0.1 cm in duplicate. We calculated maternal adiposity using the sum of skinfold thicknesses (bicep, triceps, and subscapular skinfold thickness), measured using Holtain skin calipers (Holtain, U.K.). We recorded three readings to the nearest 0.2 mm, using techniques described by Tanner and Whitehouse.^20^

Additionally, we used OGTT values to diagnose gestational diabetes mellitus between 24 and 36 weeks of gestation. We calculated gestational age based on the report of the ultrasound scan. If the ultrasound scan report was unavailable, we used a medical record or self-reported date of the last menstrual period to ascertain the gestational age. Women also completed the Edinburgh Postnatal Depression Scale during pregnancy as a measure of prenatal depression.^21,22^ At 1 year, we noted the duration of breastfeeding and classified the duration of breastfeeding as ≤6 or >6 months.

### Statistical analysis

Among the 638 infants included in the analysis for the primary outcomes at 1 year of age, there were two key covariates with missing data: birth weight (*n* = 164 missing) and maternal adiposity (*n* = 17 missing). Little’s Missing Completely at Random (MCAR) test was performed to assess whether data were missing at random or non-random.^23^ We performed Multiple Imputation by Chained Equations (MICE) method using linear regression model with 20 imputation data sets in R version 3.6.3 using MICE package.^24^ The variables included in the MICE model were maternal age, religion, education status, socioeconomic status, parity, depression status, diabetes status, delivery type, infant sex, duration of breastfeeding, and gestational age. Inverse probability weighting (IPW) method was performed in STATA 14 to estimate the probability of loss to follow-up and to use the inverse probability of these weights to determine whether loss to follow-up influenced our results. The variables used in the IPW model were maternal age, religion, education status, socioeconomic status, parity, depression status, diabetes status, duration of breastfeeding, infant sex and gestational age, for which we had complete data for the participants who were followed up.

We performed multivariable linear regression models in STATA 14 to assess the association of delivery mode with BMI *z* and length *z*. We then used multivariable Poisson regression with a robust variance to assess the association of delivery mode with infant overweight (BMI *z* ≥85th percentile) at 1 year of age. In multivariable regression models, we included maternal age, religion, education, socioeconomic status, parity, depression, height, adiposity, gestational diabetes status, infant gestational age, birth weight, and duration of breastfeeding. The models with and without multiple imputation are presented, as are the models with and without IPW.

### Ethics approval

The study was reviewed and approved by the IEC of the Indian Institute of Public Health–Bengaluru campus vide IEC no. IIPHHB/TRCIEC/091/2015 dated 13 July 2015 and IEC no. IIPHHB/TRCIEC/121/2017 dated 24 July 2017. Informed consent before participation in the study was obtained from all participants for participation, follow-ups, and permission to publish anonymous data in any report or journal.

## Results

A total of 638 mother–infant pairs were included in the analytic sample for the primary outcomes at 1 year of age. The mean age of mothers at enrollment was 24.1 years. Among the 638 women, 51.7% belonged to the Islamic religion, 66.0% were from lower socioeconomic status, 97.5% were literate, 15.1% were diagnosed with gestational diabetes, and 10.7% had an Edinburgh Postnatal Depression Scale score >13. The mean gestational age at delivery was 38.6 (SD: 1.5) weeks. Out of 638 women, 16.9% had undergone elective C-section, 26.5% had emergency C-section, and 56.6% had a spontaneous vaginal delivery. The mean age was 25.3 years for women undergoing elective C-section compared to 24.1 years for women undergoing vaginal delivery. The median maternal adiposity score, determined by skinfolds, was higher (47.8 mm) for women who underwent elective C-section compared to women who underwent vaginal delivery (43.1 mm).

Of the 638 infants in the analytic sample, 50.6% were females. The median infant age at the 1 year visit was 394.5 days (interquartile range: 373.7, 433.0 days). The mean birth weight was 2.75 (SD: 0.36) kg, BMI *z* at 1 year was −0.45 (SD: 1.53) and length *z* at 1 year was −1.12 (SD: 1.71). Most mothers (91.5%) breastfed for >6 months (Table 1). The characteristics of women who were not reachable through phone call or in-person visits at 1 year of age (*n* = 333) are presented in etable 2.

**Table 1:** Distribution of maternal and infant characteristics by delivery mode in the MAASTHI cohort (2016–2019).

	Categories	Total (*n* = 638)	Vaginal delivery (*n* = 361)	Emergency C-section (*n* = 169)	Elective C-section (*n* = 108)
*Maternal characteristics*					
Maternal age in years, mean ± SD		24.13 ± 4.00	24 ± 3.86	23.64 ± 4.24	25.3 ± 3.87
Religion, *n* (%)	Hindu	294 (46.08%)	157 (43.49%)	84 (49.70%)	53 (49.07%)
	Islam	330 (51.72%)	198 (54.85%)	81 (47.93%)	51 (47.22%)
	Others^a^	14 (2.19%)	6 (1.66%)	4 (2.37%)	4 (3.70%)
Socioeconomic status, *n* (%)	Lower SES	421 (65.99%)	251 (69.53%)	93 (55.03%)	77 (71.30%)
	Upper SES	217 (34.01%)	110 (30.47%)	76 (44.97%)	31 (28.70%)
Education, *n* (%)	Illiterate	16 (2.51%)	9 (2.49%)	4 (2.37%)	3 (2.78%)
	Up to middle school	425 (66.61%)	254 (70.36%)	94 (55.62%)	77 (71.3%)
	Above middle school^b^	197 (30.88%)	98 (27.15%)	71 (42.01%)	28 (25.93%)
Parity, *n* (%)	Nulliparous	291 (45.61%)	152 (42.11%)	124 (73.37%)	15 (13.89%)
	Primiparous	284 (44.51%)	160 (44.32%)	40 (23.67%)	84 (77.78%)
	Multiparous	63 (9.87%)	49 (13.57%)	5 (2.96%)	9 (8.33%)
Gestational diabetes, *n* (%)	Non-GDM	542 (84.95%)	319 (88.37%)	138 (81.66%)	85 (78.70%)
	GDM	96 (15.05%)	42 (11.63%)	31 (18.34%)	23 (21.30%)
Prenatal depression (EPDS score), *n* (%)	≤13	570 (89.34%)	322 (89.2%)	151 (89.35%)	97 (89.81%)
	>13	68 (10.66%)	39 (10.80%)	18 (10.65%)	11 (10.19%)
Body mass index (BMI), mean ± SD		24.24 ± 4.20	23.68 ± 4.21	24.57 ± 3.85	25.61 ± 4.38
Adiposity in mm, median (IQR)		45.1 (35.9, 68.5)	43.1 (34.4,52.9)	46.55 (37, 56)	47.8 (38.45, 56.55)
*Infant characteristics*					
Sex, *n* (%)	Male	315 (49.37%)	175 (48.48%)	85 (50.30%)	55 (50.93%)
	Female	323 (50.63%)	186 (51.52%)	84 (49.70%)	53 (49.07%)
Age at 1 year in days, median (IQR)		394.50 (373.75, 433.00)	393.00 (373.00, 429.00)	397.00 (374.00, 437.00)	393.00 (374.00, 433.5)
Birth weight in kg, mean ± SD		2.75 ± 0.36	2.77 ± 0.36	2.73 ± 0.34	2.71 ± 0.36
Gestational age in weeks, mean ± SD		38.64 ± 1.49	38.7 ± 1.48	38.89 ± 1.46	38.09 ± 1.44
BMI for age *z*-score at 1 year, mean ± SD		−0.45 ± 1.53	−0.64 ± 1.44	−0.32 ± 1.57	−0.03 ± 1.67
Length for age *z*-score at 1 year, mean ± SD		−1.12 ± 1.71	−1.08 ± 1.59	−0.94 ± 1.81	−1.53 ± 1.87
Duration of breastfeeding, *n* (%)	<6 months	54 (8.46%)	33 (9.14%)	16 (9.47%)	5 (4.63%)
	≥6 months	584 (91.54%)	328 (90.86%)	153 (90.53%)	103 (95.37%)

*n* = sample size; sample size was *n* = 638 for all variables save for maternal BMI (*n* = 621), maternal adiposity (*n* = 621), and birth weight (*n* = 474).

*SD* standard deviation, *IQR* interquartile range, *EDPS* Edinburgh Postnatal Depression Scale.

^a^Includes Christianity, Jainism, and atheist.

^b^Includes those who completed pre-university, graduation, and post-graduation; maternal adiposity and infant adiposity were defined based on the sum of skinfold thickness.

### Missing data analysis

Little’s MCAR test provided statistical evidence to suggest missingness was at random (*χ*^2^ = 44.3 df = 32, *p* value = 0.08). Multiple imputation results for birth weight in kgs, maternal adiposity in mm, and maternal BMI by delivery mode is shown in etable 1.

The results of multivariable linear regression models (with and without imputation) are shown in Table 2. Infants delivered by elective C-section had a 0.57 unit (95% confidence interval (CI) 0.20, 0.95) higher BMI *z* compared to infants that were vaginally delivered after adjusting for covariates, and this finding was similar after imputation (*β* = 0.58, 95% CI 0.24, 0.91). Emergency C-section, on the other hand, was not associated with infant BMI *z* score (*β* = 0.20, 95% CI −0.13, 0.53) (Table 2). Neither elective (*β* = −0.17, 95% CI −0.56, 0.25) or emergency (*β* = 0.12, 95% CI −0.26, 0.50) C-section was associated with infant length *z* at 1 year before multiple imputation. Yet, elective C-section delivery was associated with reduced linear growth at 1 year after multiple imputation (*β* = −0.38, 95% CI −0.76, −0.01) (Table 2).

**Table 2:** Multivariable adjusted^a^ linear regression association of delivery mode with infant BMI *z* and length *z* at 1 year of age.

Mode of delivery	Without multiple imputation^b^		With multiple imputation^b^	
	BMI *z*	Length *z*	BMI *z*	Length *z*
	*β* (95% CI)	*β* (95% CI)	*β* (95% CI)	*β* (95% CI)
Vaginal delivery (reference)	1 (reference)	1 (reference)	1 (reference)	1 (reference)
Emergency C-section	0.20 (−0.13, 0.53)	0.12 (−0.26, 0.50)	0.25 (−0.04, 0.54)	0.09 (−0.24, 0.42)
Elective C-section	0.57 (0.20, 0.95)	−0.17 (−0.56, 0.25)	0.58 (0.24, 0.91)	−0.38 (−0.76, −0.01)

^a^Multivariable adjusted models include: mothers age, religion, education, socioeconomic status, parity, depression, mother’s BMI, mother’s adiposity, gestational diabetes status, infant gestational age, and infant birth weight.

^b^The sample size without imputation was *n* = 248 for vaginal delivery, *n* = 129 for emergency C-section, and *n* = 84 for elective C-section, whereas the sample size with imputation was *n* = 361 for vaginal delivery, *n* = 169 for emergency C-section, and *n* = 108 for elective C-section.

Imputation was done for maternal BMI and adiposity and infant birth weight.

The results of multivariable Poisson regression models (with and without imputation) are shown in Table 3. Among the 638 infants, 14.9% were classified as being overweight (BMI *z* ≥85th percentile) at 1 year of age. Among infants delivered by elective C-section (*n* = 108), 25% became overweight. Compared to vaginally delivered infants, infants delivered by elective C-section had 2.34 times the risk of being overweight before adjusting covariates (95% CI 1.35, 4.05) (Table 3). After adjusting for covariates, infants delivered by elective C-section had 2.44 (95% CI 1.35, 4.41) times the risk of being overweight compared to those born by vaginal delivery. The multivariable-adjusted risk ratio (RR) was similar after multiple imputation (RR = 2.25, 95% CI 1.42, 3.57). On the other hand, emergency C-section was not associated with the risk of infant overweight (RR = 1.16, 95% CI 0.71, 1.90) (Table 3). The results of IPW analysis are presented in etables 3 and 4 and indicate no major deviations from the results in the main analyses.

**Table 3:** Multivariable^a^ adjusted Poisson regression association of delivery mode with infant overweight (BMI-for-age *z*-score ≥85th percentile) at 1 year of age.

Mode of delivery	No. of overweight infants (%)	Unadjusted RR (95% CI)	Without multiple imputation	With multiple imputation^b^	
			Adjusted RR (95% CI)	No of Overweight infants (%)	Adjusted RR (95% CI)
Vaginal delivery	42 (44.21%)	1 (reference)	1 (reference)	42 (11.63%)	1 (reference)
Emergency C-section	26 (27.37%)	1.04 (0.55, 1.98)	0.94 (0.47, 1.86)	26 (15.38%)	1.16 (0.71, 1.90)
Elective C-section	27 (28.42%)	2.34 (1.35, 4.05)	2.44 (1.35, 4.41)	27 (25.00%)	2.25 (1.42, 3.57)

^a^Multivariable adjusted models include: maternal age, religion, education, socioeconomic status, parity, depression, BMI, adiposity, and gestational diabetes status, and infant gestational age and birth weight.

^b^The sample size without imputation was *n* = 248 for vaginal delivery, *n* = 129 for emergency C-section, and *n* = 84 for elective C-section, whereas the sample size with imputation was *n* = 361 for vaginal delivery, *n* = 169 for emergency C-section, and *n* = 108 for elective C-section.

## Discussion

In our prospective birth cohort from India, infants born by elective C-section had higher BMI *z*-scores and a greater risk of overweight at 1 year of age compared to vaginally delivered infants. Infants delivered by emergency C-section, on the other hand, did not have significantly higher BMI *z*-scores or higher risk of overweight at 1 year of age. Neither elective nor emergency C-section was associated with linear growth at 1 year before multiple imputation, but after imputation elective C-section was associated with reduced linear growth. Although several studies in developed countries have now examined delivery mode and offspring risk of overweight and obesity, to our knowledge our study is the first in a less-developed country to examine delivery mode with infant growth while differentiating the type of C-section. This is an important consideration because elective and non-elective (i.e., emergency) C-sections may differ in terms of mother-to-newborn microbial transmission^25–27^ and fetal distress,^28^ factors that could be associated with later obesity.

Our finding that elective C-section, but not emergency C-section, is associated with a higher risk of infant overweight is consistent with a recent birth cohort study which found that elective C-section was associated with higher odds of infant overweight at 1 year (odds ratio (OR) 2.01, 95% CI 1.13–3.58), but emergency C-section was not (OR 1.08, 95% CI 0.66–1.76).^29^ In the Nurture study, we found that C-section infants had higher weight-for-length *z*-scores and sum of subscapular and triceps skinfold thickness when they were 1 year of age.^5^ Two Copenhagen cohorts also showed that C-section-delivered infants had higher mean BMI *z* at 6 months compared to those delivered vaginally.^30^ But these studies did not differentiate by type of C-section. Two other studies that did stratify by type of C-section did not find evidence that type of C-section modified risk for overweight and obesity when offspring were 2 and 5 years of age^28,31^ or adults.^32^ Divergent results may be due to differences in exposure classification (see below), differences in the population exposure levels (see below), or because results do not vary by C-section type. Large-scale longitudinal birth cohort studies stratifying on type of C-section delivery and whether the delivery was preceded by labor or rupture of membranes are needed to determine whether all types of C-section have similar implications for offspring health outcomes. Studies are also needed to shed light on whether mechanisms are related to mother–newborn microbiota transmission, differential stress response to labor,^5^ or delivery-mode-induced epigenetic programming.^6^

Our outcome is at 1 year of age, but the findings may apply to later stages in the life course. A systematic review by Kuhle et al. showed a pooled RR of 1.34 (95% CI 1.18–1.51) for obesity in children delivered via C-section compared with vaginal birth across ages 2–18 years.^33^ Meta-analysis studies have shown that C-section leads to mean BMI difference of 0.44 kg/m^2^ (0.17, 0.72; *p* = 0.002) with an adjusted pooled OR of 1.50 (95% CI 1.02, 2.20, *I*^2^ = 74%).^34,35^ Our results also concur with findings from birth cohort studies in the United Kingdom,^28,30,36^ China,^37^ Jerusalem,^38^ Netherlands,^39^ and North America.^40–44^ Nevertheless, a recent study of Swedish adult men, which did not have anthropometric data before adulthood, suggested that the association between C-section and overweight and obesity may not persist into adulthood.^32^

To our knowledge, there is a paucity of studies on the potential effects of elective or emergency C-section on infant linear growth. This investigation is justified because C-section infants have a greater prevalence of opportunistic pathogens,^45,46^ and elective C-section has been associated with a greater risk of infectious morbidity^47^ and respiratory morbidity in previous studies.^48,49^ In a Brazil birth cohort, which did not stratify by type of C-section, the height of the child at 6 years of age was not associated with C-section after adjusting for socioeconomic status.^50^ In our study, elective C-section, but not emergency C-section, was associated with reduced linear growth at 1 year of age in the multiple imputation model. Our findings thus provide some evidence that elective C-section result in slightly restricted postnatal linear growth. Larger birth cohort studies are needed to confirm these findings by type of C-section delivery.

There are several reasons why our findings may differ from previous studies. Initial postnatal acquisition of microbiota differs dramatically by geography, culture, and even hospital^16^ in which the infant was delivered. For these reasons, it is essential to address this research question in diverse cohorts from around the world. There may also be differences in the manner in which emergency vs elective C-sections were classified in the charts and the percentage of emergency C-sections that had spontaneous labor with rupture of membranes. Also, the association of C-section with offspring BMI *z*-score may vary over the lifecycle. Our study examined differences until 1 year of age, while other cohorts examined offspring in adulthood.

### Strengths of the study

There are several strengths to our study. First, our study is a prospective birth cohort from a less-developed country and one of the first in such a setting to address the influence type of C-section on infant growth. Our research team collected clinical, socio-demographic, and anthropometric variables that we were able to use in multivariable regression models to account for potential confounding and model effect measure modification. When information on variables was missing, we used multiple imputation, based on the pattern of missing for each variable, to check the bias in complete case analysis. Another strength is that we had a direct, standardized, and validated measurement of anthropometry both in pregnant women and in children. To reduce inter-observer and intra-observer variation of these measures, we had research assistants who were well trained and certified on an annual basis.

### Limitations

Our study also had some limitations. First, we did not have information on whether the woman was in labor or had ruptured membranes. Second, our primary outcome was assessed at 1 year of age, and it is unclear what the trajectory would be if we had multiple weights and heights within the first year of life. Third, although we adjusted for prenatal potential confounders and breastfeeding, we cannot rule out the potential influence of unmeasured factors, including unmeasured postnatal environmental factors that could have mediated or modified the association of delivery mode with infant growth at 1 year of age. Fourth, we did not have biospecimens to examine whether the observed associations were due to changes in the microbiome or other factors. Fifth, since the cohort comprised women and children from low- and middle-income groups, our results may not generalize to other socioeconomic groups in this population. Sixth, we had a limited number of infants with BMI *z*-score  ≥85th percentile. Thus we cannot rule out that the findings related to this outcome were due to chance. Finally, while BMI *z*-score is a reliable measure of excess body mass in infants, it should be noted that high BMI *z*-score at 1 year of age does not necessarily portend risk for high BMI *z*-score or overweight and obesity. However, recent data from two independent longitudinal birth cohorts show that from 6 to 24 months BMI *z*-score performed as well, if not better, than weight for length *z*-score for predicting cardiometabolic disease outcomes in adolescence.^51^

## Conclusion

In our birth cohort from India, elective but not emergency C-section delivery was associated with higher infant BMI *z*-score and a greater risk of infant overweight at 1 year of age. Furthermore, elective C-section delivery was associated with reduced linear growth but only in multiple imputation analyses. Collectively, our results suggest that the growth-promoting effects of delivery mode may be restricted to fat mass. Future research is needed to confirm our findings on linear growth and to examine whether alterations in neonatal gut microbiota or other factors underlie the observed associations. From a public health perspective, decreasing unnecessary C-sections might be a potential means to help reduce the double burden of overweight and undernutrition in children from LMICs.

## Supplementary information

Supplementary Information
