# Supplementary material for: Delivery mode and altered infant growth at 1 year of life in India
Source: Pediatr Res. 2021 Mar 2;90(6):1251–7. doi: 10.1038/s41390-021-01417-6 (PMC8671090; doi:10.1038/s41390-021-01417-6)
Supplement: Supplementary file 1 — Supplementary Information [file 41390_2021_1417_MOESM1_ESM.docx]

**SUPPLEMENTARY TABLES**

**eTable1.** Distribution of maternal and infant characteristics by delivery mode after multiple imputation.

| **Variables** | **Total (n=638)** | **Vaginal delivery**  **(n=361)** | **Emergency C-section**  **(n=169)** | **Elective C-section**  **(n=108)** |
| --- | --- | --- | --- | --- |
| **Maternal BMI**, mean ± SD | 24.65±3.92 | 24.23±4.16 | 24.79±3.48 | 25.83 ±4.22 |
| **Maternal a**diposity in mm, median (IQR) | 45.20 (36.10, 54.50) | 43.90  (34.50,52.70) | 46.3  (37.10,55.90) | 47.05  (38.65,56.15) |
| Birthweight in kg, mean ± SD | 2.74±0.31 | 2.76±0.31 | 2.74±0.3 | 2.71±0.32 |
| *SD: Standard deviation; n=sample size; IQR: interquartile range* | | | | |

**eTable 2.** Distribution of maternal and infant characteristics of those who were loss to follow up with respect to delivery mode.

|  | **Categories** | **Total (n=333)** | **Vaginal delivery**  **(n=159)** | **Emergency C-section**  **(n=101)** | **Elective C-section**  **(n=73)** |
| --- | --- | --- | --- | --- | --- |
| *Maternal Characteristics* | | | | | |
| Maternal age in yrs, mean ± SD |  | 24.05±3.88 | 23.99±3.61 | 23.58±4.18 | 24.84±3.94 |
| Religion, n (%) | Hindu | 137(41.14%) | 60(37.74%) | 44(43.56%) | 33(45.21%) |
|  | Islam | 186(55.86%) | 94(59.12%) | 54(53.47%) | 38(52.05%) |
|  | Others^#^ | 10(3.00%) | 5(3.14%) | 3(2.97%) | 2(2.74%) |
| Socioeconomic status, n (%) | Lower SES | 220(66.07%) | 110(69.18%) | 61(60.4%) | 49(67.12%) |
|  | Upper SES | 113(33.93%) | 49(30.82%) | 40(39.6%) | 24(32.88%) |
| Education, n (%) | Illiterate | 10(3.00%) | 9(5.66%) | 1(0.99%) | 0(0%) |
|  | Up to middle school | 228(68.47%) | 105(66.04%) | 69(68.32%) | 54(73.97%) |
|  | Above middle school* | 95(28.53%) | 45(28.3%) | 31(30.69%) | 19(26.03%) |
| Parity, n (%) | Nulliparous | 132(39.64%) | 53(33.33%) | 67(66.34%) | 12(16.44%) |
|  | Primiparous | 168(50.45%) | 80(50.31%) | 31(30.69%) | 57(78.08%) |
|  | Multiparous | 33(9.91%) | 26(16.35%) | 3(2.97%) | 4(5.48%) |
| Gestational diabetes, n (%) | Non-GDM | 270(81.08%) | 133(83.65%) | 78(78%) | 59(80.82%) |
|  | GDM | 62(18.62%) | 26(16.35%) | 22(22%) | 14(19.18%) |
| Prenatal depression (EPDS score^@^), n (%) | ≤ 13 | 300(90.09%) | 140(88.61%) | 96(95.05%) | 64(87.67%) |
|  | > 13 | 32(9.61%) | 18(11.39%) | 5(4.95%) | 9(12.33%) |
| Body mass index (BMI), mean ± SD |  | 24.65±4.46 | 23.54±3.97 | 25.73±4.15 | 25.53±5.31 |
| Adiposity in mm, median (IQR) |  | 46.17  (36.80,57.47) | 43.2  (33.37,55.50) | 47.53  (39.75,60.32) | 51.10  (40.13,62.45) |
| ***Infant characteristics*** | | | | | |
| Sex, n (%) | Male | 174(52.30%) | 79(49.69%) | 57(56.44%) | 38(52.05%) |
|  | Female | 159(47.7%) | 80(50.31%) | 44(43.56%) | 35(47.95%) |
| Birthweight in kg, mean ± SD |  | 2.75±0.40 | 2.73±0.36 | 2.81±0.39 | 2.71±0.48 |
| Gestational age in weeks, mean ± SD |  | 38.55±1.47 | 38.72±1.43 | 38.59±1.61 | 38.14±1.26 |
| SD: Standard deviation; n=sample size; IQR: interquartile range  # Includes Christianity, Jainism, and atheist,  *Includes those who completed pre-university, graduation, and post-graduation; Maternal adiposity and infant adiposity were defined based on the sum of skinfold thickness,  @ Edinburgh Postnatal Depression Scale  Sample size was n = 333 for all variables save for maternal BMI (n=322) and maternal adiposity (n=324) | | | | | |

**eTable 3.** Multivariable adjusted* linear regression association of delivery mode with infant BMI z and length z at one year of age adjusted for IPW analysis.

|  | Without multiple imputation^†^ | | With multiple imputation^†^ | |
| --- | --- | --- | --- | --- |
| Mode of delivery | **BMI z** | **Length z** | **BMI z** | **Length z** |
|  | β (95% CI) | β (95% CI) | β (95% CI) | β (95% CI) |
| Vaginal delivery (reference) | 1 (reference) | 1 (reference) | 1 (reference) | 1 (reference) |
| Emergency C-section | 0.19(-0.14,0.52) | 0.14(-0.24,0.51) | 0.24(-0.05,0.53) | 0.10(-0.22,0.43) |
| Elective C-section | 0.58(0.20,0.95) | -0.18(-0.60,0.25) | 0.57(0.24,0.91) | -0.38(-0.76, -0.01) |
| *Multivariable adjusted models include: mothers age, religion, education, socio economic status, parity, depression, mother’s BMI, mother’s adiposity, gestational diabetes status, infant gestational age, and infant birthweight.  ^†^The sample size without imputation was n=248 for vaginal delivery, n=129 for emergency C-section, and n=84 for elective C-section, whereas the sample size with imputation was n=361 for vaginal delivery, n=169 for emergency C-section, and n=108 for elective C-section.  Imputation was done for maternal BMI, adiposity, and birth weight | | | | |

**eTable 4.** Multivariable* adjusted Poisson regression association of delivery mode with infant overweight (BMI-for-age Z score ≥85^th^ percentile) at one year of age adjusted for IPW analysis.

| **Mode of delivery** | **No. of overweight infants (%)** | **Unadjusted IPW RR (95% CI)** | Without multiple imputation | With multiple imputation^†^ | |
| --- | --- | --- | --- | --- | --- |
|  |  |  | **Adjusted IPW RR (95% CI)** | **No of Overweight infants (%)** | **Adjusted IPW RR (95% CI)** |
| **Vaginal delivery** | 24 (9.68%) | 1 (reference) | 1 (reference) | 42 (11.63%) | 1 (reference) |
| **Emergency C-section** | 13 (10.08%) | 1.35 (0.86, 2.14) | 0.94(0.47,1.86) | 26 (15.38%) | 1.97(1.70,2.28) |
| **Elective C-section** | 19 (22.62%) | 2.11 (1.37, 3.25) | 2.53(1.40,4.59) | 27 (25.00%) | 2.27(1.96,2.64) |
| *Multivariable adjusted models include: mothers age, religion, education, socio economic status, parity, depression, mother’s BMI, mother’s adiposity, gestational diabetes status, infant gestational age, and infant birthweight**.**  ^†^The sample size without imputation was n=248 for vaginal delivery, n=129 for emergency C-section, and n=84 for elective C-section, whereas the sample size with imputation was n=361 for vaginal delivery, n=169 for emergency C-section, and n=108 for elective C-section. | | | | | |
